# Antibacterial effectiveness of multi-strain probiotics supernatants intracanal medication on *Enterococcus faecalis* biofilm in a tooth model

**DOI:** 10.1186/s12903-023-02914-2

**Published:** 2023-04-20

**Authors:** Shymaa Shaaban, Salma Genena, Alaaeldin Elraggal, Gamal M. Hamad, Marwa A. Meheissen, Sybel Moussa

**Affiliations:** 1 Division of Endodontics, Conservative Dentistry Department, Faculty of Dentistry, Alexandria University, Alexandria, Egypt; 2 Division of Operative Dentistry, Conservative Dentistry Department, Faculty of Dentistry, Alexandria University, Alexandria, Egypt; 3 Department of Food Technology, Arid Lands Cultivation Research Institute (ALCRI), City of Scientific Research and Technological Applications (SRTACity), New Borg El-Arab, Alexandria, Egypt; 4 Medical Microbiology & Immunology Department, Faculty of Medicine, Alexandria University, Alexandria, Egypt

**Keywords:** Probiotics, *Lactobacillus*, Intracanal medication, *Enterococcus faecalis* biofilm, Calcium hydroxide

## Abstract

**Background:**

To assess the antibacterial activity of multi-strain probiotics supernatants (MSP); *Lactobacillus plantarum*, *Lactobacillus rhamnosus*, and *Lactobacillus acidophilus* as an intracanal medication on *Enterococcus faecalis* (*E. faecalis*) biofilm in a tooth model.

**Methods:**

Sixty extracted human single-rooted teeth with single canals were instrumented, sterilized, and inoculated with *E. faecalis*. After 21 days of incubation, four specimens were randomly selected to validate the biofilm formation by scanning electron microscope (SEM). The remaining specimens were randomly divided (n = 14), according to the intracanal medication (ICM) received into: **Ca(OH)**_**2**_: calcium hydroxide paste (35% Ultra Cal XS Ca(OH)_2_), **Probiotics supernatants**: MSP in poloxamer gel vehicle **Poloxamer**: poloxamer gel vehicle and, **Control**: *E. faecalis* biofilm only. The tested groups were further subdivided into two equal subgroups (n = 7) according to the incubation period (24 h and 7 days). Shaved dentin chips were obtained and collected by H-files and paper points, respectively for bacterial culture. The antibacterial activity was assessed after each incubation period quantitatively and qualitatively using bacterial colony-forming units per milliliter (CFUs/ml) and SEM, respectively.

**Results:**

The lowest CFUs/ml was found in Ca (OH)_2_ with a significant difference compared to other groups after 24 h. After 7 days, a similar outcome was found with a further significant reduction of CFUs/ml in all groups with no statistical difference between Ca(OH)_2_ and probiotics supernatants groups. Ca (OH)_2_ and Probiotics supernatants groups showed a significant (p < 0.05) percentage of overall bacterial reduction (100.00 ± 0.00% and 70.30 ± 12.95%, respectively) compared to poloxamer and control groups (27.80 ± 14.45 and 28.29 ± 19.79). SEM images showed a bacteria-free state in the Ca(OH)_2_ group after 7 days while few bacteria were found in the probiotics supernatants group. An extensive invasion of bacteria was found in poloxamer and controls groups.

**Conclusion:**

MSP has a potential antibacterial effect on *E. faecalis* growth closely similar to the routinely used Ca (OH)_2_.

## Background

Apical periodontitis (AP) is the inflammation of peri-radicular tissues secondary to persistent bacterial infection in the dental pulp [1]. In necrotic pulps, microbes grow, aggregate, and populate the pulp space ending up with the invasion and irritation of the peri-radicular tissues [2]. Successful root canal treatment (RCT) depends, to a great extent, on the eradication or significant decrease of the bacterial load and their toxins down to the healing capacity of the periodontal tissues [3]. Mechanical instrumentation and chemical disinfection, using irrigants and intracanal medicaments, are the routine approaches followed by clinicians to non-specifically kill the microbial load of the invaded pulp system [4]. A positive outcome of RCT is verified by the healing of peri-radicular tissues as indicated by the follow-up radiographs [2]. However, the substandard quality of RCT might render the pulp space with infectious bacteria [5, 6]. Missed canals, poor chemo-mechanical root canal preparation, improper access cavity design, and inadequate coronal seal are further potential causes for persistent microbial infection and hence, a non-resolving AP [6].

Intracanal medications have been routinely used, in conjunction with the chemo-mechanical root canal preparation, to reach a satisfactory control on eradication of the microbial load in pulp space. Calcium hydroxide (Ca(OH)_2_) is the commonly used intracanal medicament (ICM) for the treatment of AP due to its antibacterial effect [7, 8]. It can be used alone or coupled with 2% chlorhexidine to increase its efficacy against resistant bacteria [9, 10]. *Enterococcus faecalis* (*E. faecalis*) is the most consistently reported microorganism in cases with AP. It is characterized by the ability to survive extreme environmental conditions; such as high alkalinity with a pH of up to 11.5. It can, therefore, be resistant to Ca(OH)_2_ ICM by maintaining its internal pH through a proton pump mechanism [11]. Further, it can survive a significantly long period of starvation and can grow solely to cause infection with no need for other synergistic microbes. Therefore, *E. faecalis* can be considered the most unmanageable microbe in cases of AP. Ca(OH)_2_ has other limitations such as; the need for operator skills to place the Ca(OH)_2_ into posterior teeth with a complex root canal anatomy, and the difficulty of completely removing it rendering the pulp canals with residual Ca(OH)_2_. Remnants of Ca(OH)_2_ can interfere with the setting reaction of ZnO eugenol-based root canal sealers [12]. Despite the widespread use of Ca(OH)_2_ in endodontics, it may still not be the best option for eliminating all bacterial pathogens in root canals. Hence, finding another alternative therapeutic ICM is essential to overcome the limitations of Ca(OH)_2_.

Probiotics are a group of living microorganisms that have evolved as an alternative therapeutic tool in the treatment of many chronic diseases [13]. They have been largely implicated in the management of gastrointestinal problems such as; chronic inflammation of the gut and irritable bowel syndrome [14]. Further implications have been extended to include the management of anxiety, depression, and autism [15]. In addition, previous studies reported a significant link between probiotics and delayed progress of Alzheimer’s disease [16]. The use of probiotics in dentistry has been reported in a few clinical studies of dental caries [17] and gum diseases [18]. *Lactobacillus* and *Bifidobacterium* strains are examples of probiotics with reported antibacterial action to *E. faecalis* in one previous study [19]. Probiotics from; *Lactobacillus plantarum (L. plantarum), Lactobacillus acidopilus*(*L. acidopilus*), and *Lactobacillus rhamnosus*(*L. rhamnosus*) reduced the growth of *E. faecalis* in vitro and ex vivo when tested separately in previous studies [20, 21].

Probitoics supernatants were previously investigated for their antimicrobial role against *E. faecalis* using agar well diffusion method [22]. Probiotics supernatants were hypothesized to be a promising alternative option to the routinely used Ca(OH)_2_ ICM against *E. faecalis*. However, due to the scarcity of data and limited studies in the literature, it is unclear to draw a definitive conclusion on how probiotics supernatants can interact with *E. faecalis* in a tooth model. Further, the use of multi-strain probiotics supernatants as a single ICM may interact more effectively against *E. faecalis*. However, this hypothetical interaction has not yet been tested and verified. Therefore, this in-vitro study aimed to investigate the effect of multi-strain probiotics supernatants (*L. plantarum, L. acidopilus*, and *L. rhamnosus*) on lab-grown *E. faecalis* biofilm in a tooth model. The study was guided by the null hypothesis that probiotics will not significantly affect the microbial load of *E. faecalis.*

## Materials and methods

### Study design and sample size calculation

**Fig. 1:**
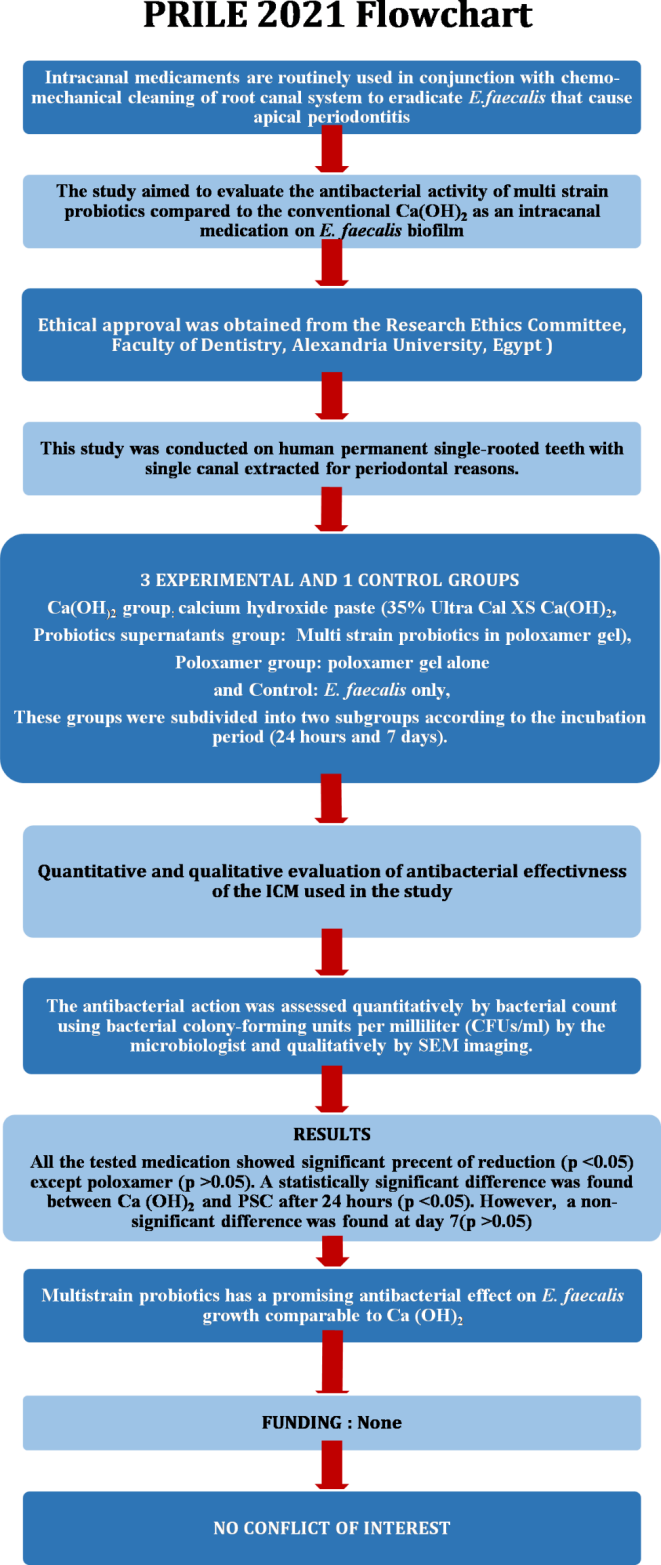
PRILE 2021 flow chart for the current study

The manuscript of this laboratory study has been written according to Preferred Reporting Items for Laboratory studies in Endodontology (PRILE) 2021 guidelines [23] (Fig. 1). Ethical approval was obtained from the Research Ethics Committee, Faculty of Dentistry, Alexandria University (IRB 00010556 – IORG 0008839). Informed consent was obtained from all subjects and/or their legal guardian(s) who voluntarily agreed to dontae their extracted teeth for the current study.

Sample size calculations were performed using MedCalc Statistical Software (MedCalc Software bvba, Ostend, Belgium; http://www.medcalc.org; 2018). The sample size was planned on a significance level of 5% α and a power of 80%. It was calculated to be 5 per group increased to 7 to compensate for any potential laboratory errors [24, 25]. The total sample size required to compare the antibacterial effectiveness of different intra-canal medications at different follow-up intervals = number of subgroups × number of specimens per subgroup = 8 × 7 = 56 [26]. One extra sample was added to each main group for biofilm validation by Scanning electron microscope (SEM). Therefore, a total of 60 freshly extracted, for periodontal involvement, and caries-free human permanent single-rooted teeth with a single canal were used in the current study. Extracted teeth were collected from the outpatient clinic of the Oral and Maxillofacial Surgery Department, Faculty of Dentistry, Alexandria University.

### Preparation of probiotics supernatants intracanal medicament

Poloxamer gel 30% (w/w) was prepared using the ‘cold technique’ under a magnetic stirrer (SCILOGEX, Rocky Hill, USA.) by adding poloxamer 407 powder (Sigma Aldrich Chemical Co., Gillingham, UK.) to sterile distilled water (4–8°C) then kept in the refrigerator for 24 h. Three strains of *Lactobacilli* Probiotics (*Lactobacillus plantarum* ATCC 14917, *Lactobacillus rhamnosus* ATCC 7469, and *Lactobacillus acidophilus* ATCC 4356) were obtained from Microbiological Resources Centre (MIRCEN). They were grown individually on deMan Rogsa Sharpe (MRS) broth (HiMedia Laboratories Pvt. Ltd, India) aerobically for 48 hours at 37°C [27]. The pure isolate of *Lactobacilli* species suspension was propagated in a 100 ml flask containing MRS broth and incubated for 72 hours at 37°C. The cell-free supernatant (CFS) was obtained by centrifuging the culture at 10,000 rpm for 10 minutes, then lyophilized using a vacuum freeze dryer (Model FDF 0350, Korea). In a previous study [27], the minimum concentration of MSP to initiate an inhibitory response against *E. faecalis* was 50 mg/ml as verified by agar well diffusion assay. Higher concentrations of MSP of 100, 200, and 300 mg/ml showed bigger zones of inhibition. In the current study, 300 mg of the obtained lyophilized CFS for each supernatant was mixed with 1ml poloxamer gel under a magnetic stirrer in an ice bath to get the MSP gel at a targeted concentration of 300 mg/ml.

### Teeth preparation

An ultrasonic scaler (Woodpecker,China.) was used to remove all calculus deposits if found, followed by ultrasonic cleaning in distilled water for 10 min and then immersed in 5.25% sodium hypochlorite for 30 min to remove soft tissue attached to the root surface. Teeth were decoronated, to a standard 14 mm root length, using a diamond disc mounted on a low-speed handpiece under water coolant. The working length (WL) was established by subtracting one millimeter from the full length of the decoronated teeth. A k-file no. 15 (MANI,Japan.) was inserted gently into the root canal at the estimated working length for radiograph taking to confirm the WL. The remaining roots were mechanically cleaned and shaped using the ProTaper Next rotary system (Dentsply Sirona Maillefer, Ballaigues, Switzerland.) up to size X3 (0.30/7%) and irrigated with 3ml of 2.5% sodium hypochlorite. The smear layer was removed by flushing the root canal with 3 ml 17% EDTA solution ( Dharma, Miami, Florida, USA) for 5 min followed by 3ml of 2.5% sodium hypochlorite. Final irrigation was employed using 5% sodium thiosulfate (PIOCHEM,Egypt) to inactivate sodium hypochlorite. The outer surfaces of the roots were covered with a double layer of nail varnish then the apical foramen of the teeth was sealed with a light-cured resin composite (Nexcomp, Dentsply Maillefer, Ballaigues, Switzerland).

Each root was individually placed in a cryovial containing 500 µl of brain heart infusion (BHI) broth (HiMedia Laboratories Pvt. Ltd, India) and autoclaved at 121° C for 30 min. Roots were then separately mounted vertically on silicon impression material (zeta plus condensation silicone, Zhermack, Badia Polesine, Rovigo, Italy) and underwent a second round of autoclave cycle to ensure the sterility of the setup. Subcultures on blood agar from randomly selected samples were obtained to verify sterilization after incubation for 48 h at 37° C.

### Cultivation and inoculation of *E. faecalis* biofilm

*E. faecalis* (ATCC29212) was grown on a blood agar-sealed petri dish at 37° C for 24 h. The colonies were suspended in 5 ml of BHI broth and adjusted by spectrophotometer to a concentration of 1 McFarland. Each root canal was inoculated with *E. faecalis* suspension using a sterile insulin syringe and incubated at 37 °C for 21 days under aseptic aerobic conditions. The inoculation was repeated every 3 days to ensure the viability of the bacteria [28, 29].

### Biofilm validation under the scanning electron microscope (SEM)

Four randomly selected specimens were sectioned longitudinally using a diamond disc, fixed by immersion in 4F1G solution (40% formalin and 25% glutaraldehyde in phosphate buffer) for 3 h at 4 °C then immersed in 2% osmium tetroxide OsO_4_ for 2 h at 4 °C, dehydrated at 4 °C through a graded series of ethanol, dried at room temperature, mounted using carbon paste on a copper stub, and sputter-coated with gold Surface roughness. Secondary electron images at 5,000 X and 10,000 X were obtained using SEM (JEOL JSM-IT200,Tokyo, Japan.) to examine and validate the formed *E. faecalis* biofilm on each root canal.

### Specimens allocation and grouping

The remaining 56 roots were dried with sterile paper points and then randomly assigned, for treatment by ICM, into four groups of 14. Groups were treated, respectively, by one of three ICM using insulin syringes: (i) **Probiotics supernatants**: MSP 300 mg/ml in poloxamer gel, (ii) **Ca(OH)**_**2**_: calcium hydroxide paste (35% Ultra Cal XS Ca(OH)_2_), (iii) **Poloxamer**: poloxamer vehicle gel, while untreated *E. faecalis*-loaded roots served as a **control group**. The coronal surface of the roots was sealed with a sterile Teflon pellet topped with temporary filling material (Ultratemp, Ultradent) and incubated at 37℃. Half of the specimens in each treated group were incubated for 1 day, while the other half was incubated for 7 successive days [25, 30].

### Assessment of antibacterial activity of ICM

At the end of each incubation period, the temporary filling material was removed and each root canal from all tested and control groups was irrigated with 6 ml of 17% EDTA followed by 6 ml of distilled water [31]. Ca(OH)_2_ groups were additionally washed with 1 ml of 0.5% citric acid solution to neutralize Ca(OH)_2_ then final irrigation with 3 ml sterile saline was done [22]. In an anaerobic workstation for bacterial culture analysis, each root was then mounted on a custom-made holder device to hold each root. At the base of the holder, a cryovial containing BHI was placed 1 cm from the root apex. A representative sample was collected by one same operator from each root canal by gentle manual scraping of dentinal walls, 1 mm shorter than WL, by Hedstrom file (H-file) #30 (MANI, Japan.) for 30 s to obtain dentin chips [30]. Dentin chips were collected by inserting three paper points of equivalent size 1 mm short of the WL and kept for 60 s. The contaminated paper points and H-files were placed into a cryovial containing 1ml BHI and were vortexed for 60 s and then 10-fold serially diluted. Sterile spreaders were used to collect 10µL from the resulting serial dilution to be immediately plated in 4 sealed blood agar petri dish, for each tested group, and incubated at 37° C for up to 24 h [22]. The antibacterial activity was then assessed quantitatively through the calculation of the Colony-forming units per milliliter (CFUs/ml) for each dish using a colony counter (Fisher Scientific, Waltham, MA, USA.) following the methods of Barbosa-Ribeiro et al. [32] and Martinho and Gomes [33]. Bacterial load reduction percentages between 24 h and 7 days were calculated following this mathematical formula [34]:

$$\frac{{CFU/ml\,at\,24\,hr - CFU/ml\,at\,7\,days}}{{CFU/ml\,at\,24\,hr}} \times 100$$

**Fig. 2:**
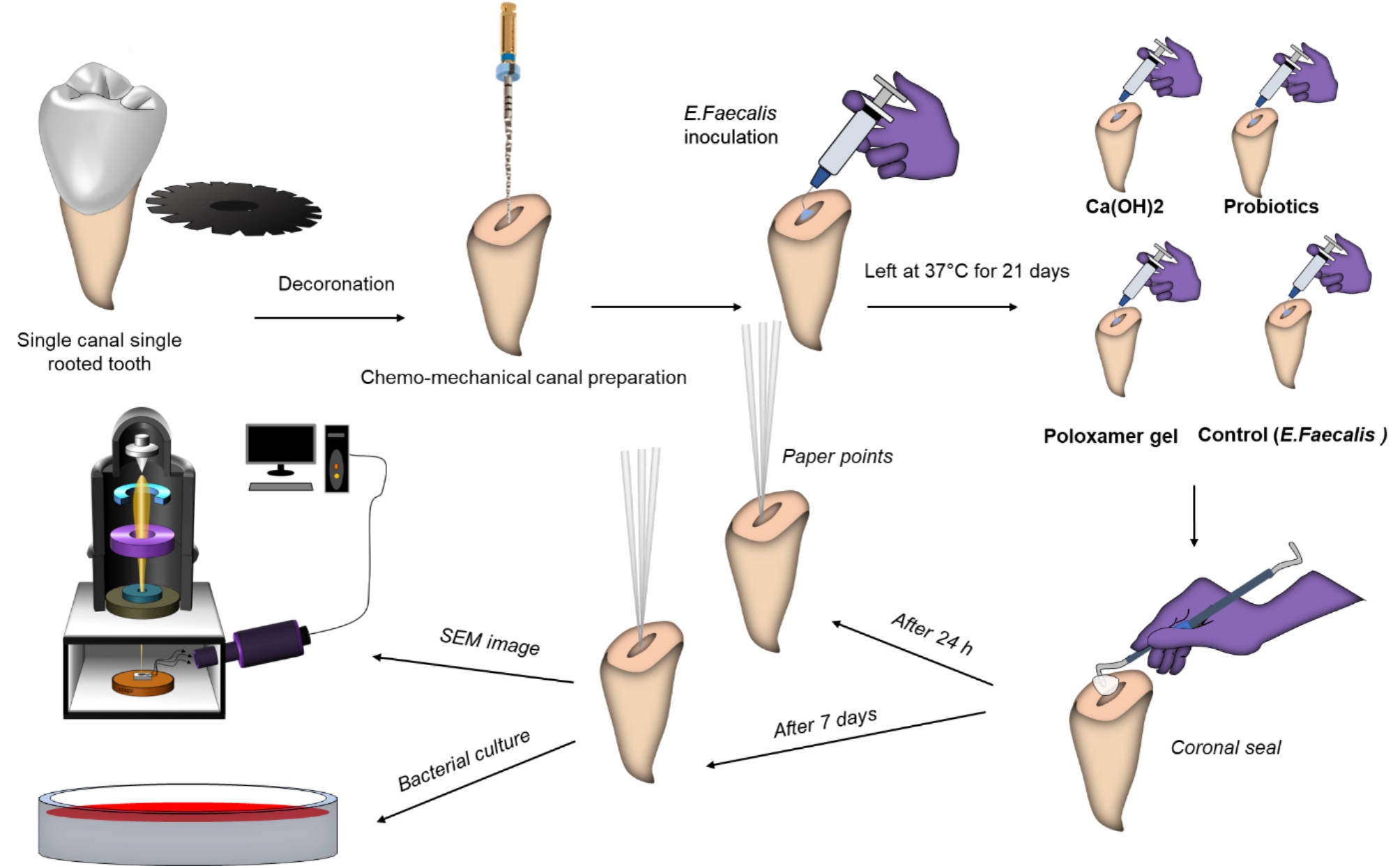
Schematic diagram showing the flow of steps of root canal preparation, E. faecalis inoculation, specimens grouping, quantitative and qualitative analysis

A further qualitative antibacterial assessment was done on the treated groups under the SEM. Two randomly treated roots from each group were longitudinally sectioned. Their apical third were dissicated, gold/palladium-coated and investigated under SEM to assess the presence of *E. faecalis* biofilm at a microscopic level (Fig. 2).

### Statistical analysis

Computer software (SPSS for Windows version 22.0 (SPSS Inc., Chicago, IL, USA)) was used to run all the statistical analyses of the obtained data. The normal distribution and homogeneity of variance were checked and verified for CFUs/ml using Kolmogorov-Smirnov and Levene’s tests, respectively. The data were found to be normally distributed. Hence, a one-way analysis of variance (ANOVA) was used to compare the mean CFU/ml of the studied groups followed by multiple pairwise comparisons using Bonferroni adjusted significance level. Comparisons of CFUs/ml of *E. faecalis* between 24 h and 7 days within each group were done using paired t-test with a significance set (p-value < 0.05).

## Results

### Biofilm validation

**Fig. 3:**
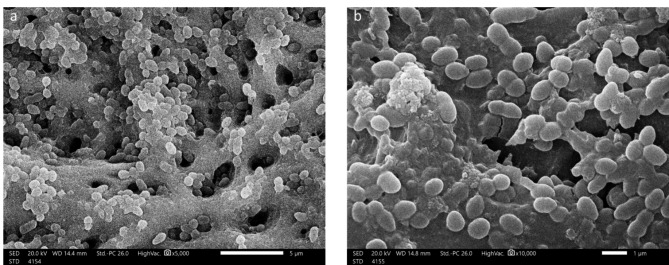
SEM images at (**a**) 5,000 X and (**b**) 10,000 X showing the formed E. faecalis biofilm on the dentinal wall of the prepared roots

The SEM analysis of the randomly selected specimens confirmed the formation of mature *E. faecalis* biofilm. The images showed that all canal walls were coated with aggregated cocci bacteria of different sizes blocking the dentinal tubules (DT) (Fig. 3).

### Assessment of antibacterial activity of ICM

**Fig. 4:**
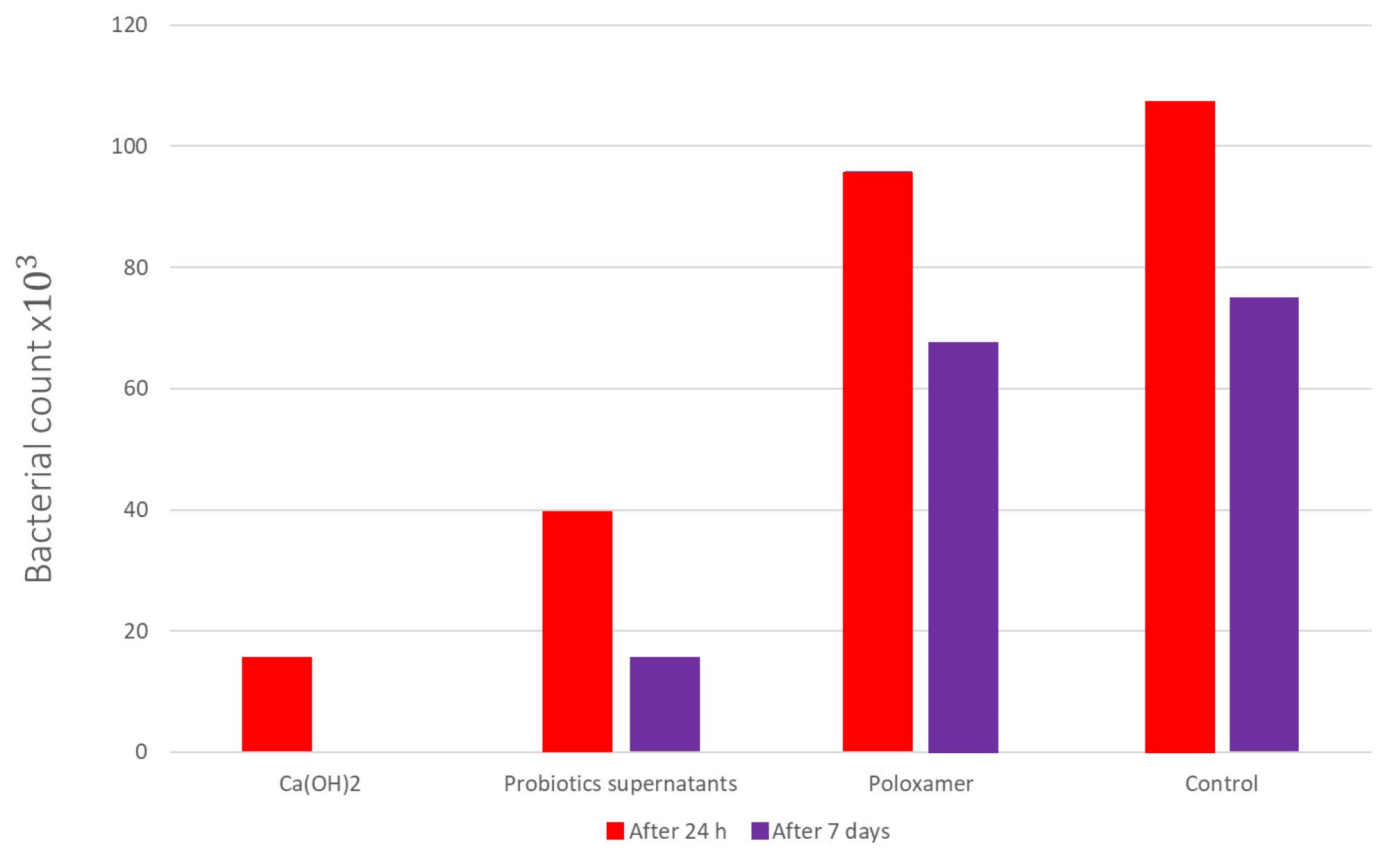
Bar charts showing the bacterial count for each group after 24 and 7 days of the intracanal medication placement

Descriptive statistics of the CFU/ml and one-way ANOVA test between the different ICMs used are presented in (Table 1) and graphically presented as bar charts in Fig. 4. After 24 h, the lowest CFU/ml was found in the Ca(OH)_2_ group followed by the Probiotics supernatants group with a statistical significance between them (p = 0.00). However, the highest CFU/ml was found in poloxamer which did not significantly differ from the control group. After 7 days, A significant reduction of CFU/ml was found in all groups with the highest percentage change of 100% in the Ca(OH)_2_ group followed by the Probiotics supernatants group (70.30 ± 12.95%), while the lowest percentage change in CFU/ml was found in Poloxamer and control groups (27.80 ± 14.45 and 28.29 ± 19.79, respectively).

**Fig. 5:**
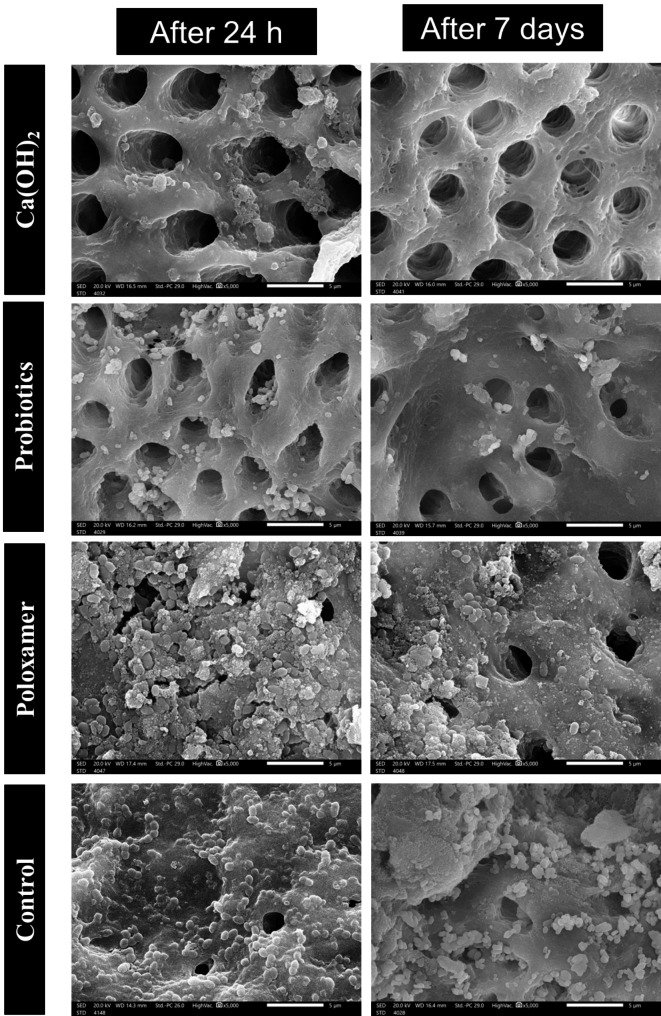
SEM images at 5,000 showing the distribution pattern of *E. faecalis* biofilm on treated pulp space at 24 h and 7 days from the application of different ICM.

SEM images at 5,000X (Fig. 5) showed few scattered *E. faecalis* biofilm on Ca (OH)_2_ and Probiotics supernatants groups with a detectable higher amount of biofilm in the latter group after 24 and 7 days, in agreement with the findings of the CFU/ml. A comparable extensive amount of *E. faecalis* biofilm and occluded dentinal tubules were observed in Poloxamer and control groups regardless of the incubation period.

**Table 1:** Descriptive statistics (mean ± SD) and percentage reduction of CFU/ml of the studied ICM between 24 h and 7 days

	CFU/ml mean ± SD		
	After 24 h	After 7 days	Percentage reduction (%)
**Ca(OH)**_**2**_	15.50 ± 5.01 × 10^3 Aa^	0.00 ± 0.00 ^Ab^	100.00 ± 0.00 ^A^
**Probiotics supernatants**	39.17 ± 11.58 × 10^3 Ba^	10.83 ± 3.43 × 10^3 Ab^	70.30 ± 12.95 ^B^
**Poloxamer**	95.83 ± 16.86 × 10^3 Ca^	67.50 ± 8.22 × 10^3 Bb^	27.80 ± 14.45 ^C^
**Control**	107.50 ± 15.41 × 10^3 Ca^	75.00 ± 14.14 × 10^3 Bb^	28.29 ± 19.79 ^C^

No statistical significance (P > 0.05) is indicated by the same superscript capital letter in columns when comparing different ICMs and by the same superscript small letters when comparing the incubation period for the same ICM

## Discussion

The current study evaluated the antibacterial effect of multi-strain probiotics supernatants on infected root canals with *E. faecalis* biofilm. A significant reduction in the bacterial count was found at 24 h and 7 days, and thus the null hypothesis was rejected.

Thorough chemomechanical cleaning and shaping of infected root canals may not be sufficient to eradicate bacterial infection [35, 36]. Hence, ICMs have been routinely used as a complementary treatment by endodontists to eradicate the bacterial load inside the infected root canal system in cases of AP. Previous studies reported a significant reduction of AP-causing bacteria by ICM [8, 35, 37]. However, other studies reported either no change or an increase in bacterial load in the infected root canal system [36, 38]. Failure of ICM might be attributed to inadequate coronal seal to the treated root canals or the complex structure of the root canal system such as apical ramifications that increase the potentiality of improper cleaning [31]. Missed canals and bacterial invasion deep into dentinal tubules are other possible causes of failure of ICM that may result in a regrowth of bacteria [6]. In the current study, however, a tight coronal seal was done by Teflon pellet topped with temporary dressing in addition to placement of the treated roots in strictly aseptic conditions to insure no bacterial contamination other than the investigated *E. faecalis*.

Ultra Cal XS was chosen as a representative example of commercially available readymade intracanal Ca(OH)_2_ medication. Ca(OH)_2_ ICM works by releasing (OH̅) ions and creating a strongly alkaline medium that kills bacteria [39, 40]. In this study, the Ca (OH)_2_ group showed significant antibacterial activity against *E. faecalis* after 24 h. and showed a higher antibacterial effect after day 7 similar to Javidi et al. [22], and Lakhani et al. [41]. It has been reported that *E. faecalis* can be an impervious strain against many ICMs including Ca(OH)_2_ [22, 39, 40, 42]. The hydroxyapatite in dentin structure can act as a buffering substrate by releasing H_2_PO_4_^−^, H_2_CO_3_^−^, and HCO_3_^–^ protons to neutralise the (OH̅) ions. This buffering capacity might reduce the pH below causing any harm to bacteria and thus rendering the ICM ineffective as agreed by previous studies [22, 42].

Probiotics are defined as “live microorganisms which when administered in adequate amounts confer a health benefit on the host” [43]. They release lactic acid, hydrogen peroxide, and bacteriocin that work together to destroy harmful bacteria. Bacteriocin is a bacterial toxin made of peptides, while hydrogen peroxide kills bacteria by destroying their cell walls [13, 44]. The antibacterial action of MSP may be attributed to the competitive exclusion of pathogens and the production of bacteriocin and other antimicrobial substances that lead to a substantial decrease in microbial load [14, 45].

Further, probiotics have a higher potential survival capacity, compared with other pathogenic bacteria, due to a competitive behavior for nutrients and space [13]. Probiotics such as *L. rhamnosus* and *L. plantarum* had an antibacterial effect when separately tested, against E. faecalis. Other studies confirmed the antibacterial effect of these probiotic species against E. faecalis and C.albicans in either planktonic or biofilm form [19]. *L. plantarum* species are capable of producing lipoteichoic acid that was found to have an antibacterial effect on *Actinomyces naeslundii, Ent. faecalis, Lact.salivarius*, and *Strep. Mutans* [46]. lipoteichoic acid is an integral part of the lactobacilli species of probiotics that has an antimicrobial effect on *E. faecalis* as reported by previous lab work [47].

With the continuous evolution of probiotics and their successful application in dentistry, it was important to test this treatment approach on the resistant *E. faecalis* species in the current study. A cumulative antimicrobial effect of the supernatants of three probiotic strains (*Lactobacillus plantarum* ATCC 14,917, *Lactobacillus rhamnosus* ATCC 7469, and *Lactobacillus acidophilus* ATCC 4356) was tested against the *E. faecalis* species biofilm. The minimum inhibitory concentration (MIC) of MSP against *E. faecalis* in a planktonic state was determined by the agar well diffusion assay in a previous pilot study to be 50 mg/ml [27]. The present study used 300 mg/ml concentration as a higher concentration than MIC to increase the antimicrobial effect against *E. faecalis* in the biofilm stage in a tooth model. Probiotics supernatants have significantly reduced *E. faecalis* biofilm after 24 h, however, Ca(OH)_2_ was significantly more effective. Despite a further significant overall reduction in *E. faecalis* biofilm by the Probiotics supernatants group after 7 days, a bacteria-free pulp canal system was reached in the Ca(OH)_2_ group. A longer contact time of MSP gel may be needed to yield better antibacterial action and reach a bacteria-free state. Similar to our results, Kim et al. [50] showed that *L. plantarum* reduced growth of *E. faecalis* in vitro and ex vivo. Additionally, Noushad et al. [20] compared the effect of *L. acidopilus* against *E. faecalis* with Ca(OH)_2_ and stated that probiotics could inhibit *E. faecalis* more than Ca(OH)_2_.

Poloxamer was selected as a delivery vehicle for the multistrain probiotics supernatants. Poloxamer gel is a thermoreversible hydrogel that undergoes in-situ gelation by heating above the lower critical gelation temperature and it is a biocompatible vehicle for drug delivery [48]. In addition, it was used as a vehicle for antibiotic delivery through pockets for the treatment of periodontal disease [48, 49]. Poloxamer vehicle showed no significant bacterial reduction, so it is a suitable vehicle for MSP, and this is in agreement with Bohora et al. [19, 59].

A three-week-old *E. faecalis* biofilm was used in a tooth model to mimic the endodontic environment. The three-week-old biofilm was anticipated to confirm the formation of a high-resistance biofilm as supported by a previous study [51] which found that three-week-old biofilm is more resistant than one and two- week- old biofilm. This is in accordance with previous works [24, 41, 52]. This study used two incubation periods; 24 h and 7 days as suggested by Javidi et al. [22]. The 24 h was used as an early incubation period as El-Sayed et al., [24] reported a reduction of *E. faecalis* immediately after irrigation with *L. rhamnosus* and after 24 h. While 7 days incubation period was selected as it is the minimum time needed for Ca(OH)_2_ to be used as an interappointment medication [53]. The complete removal of Ca(OH)_2_ is needed to stop its residual effect. Citric acid is an effective chelator agent in removing intracanal medications [54]. The minimum bactericidal concentration of citric acid against *E. faecalis* is 20%. Thus, in this study, 1 ml of 0.5% citric acid was used to rule out further antibacterial effect of any agent other than Ca(OH)_2_ on *E. faecalis*. Saline was also chosen for removal of MSP because it has no antibacterial action and it is the irrigant of choice for removal of many medicaments in in vitro studies such as chlorohexidine, triple antibiotic paste and Ca(OH)_2_ [55].

Microbiological sampling was done following Tirukkolluru et al., [56] by using Hedstrom file #30 to scrape the dentin wall to retrieve bacteria inside dentinal tubules and three sterile paper points of equivalent size were used to absorb the bacteria suspended in the root canal. Then, collected in a cryovial containing BHI and vortex for 60 s before culturing on an agar plate. CFUs/ml were used in bacterial count as it is an accurate method to detect viable bacteria and evaluate the efficacy of intracanal medication [57]. This method has been used as a reliable quantitative analysis of bacterial count in previous studies [24, 41, 58]. To validate the work, SEM analysis was performed on randomly selected samples to check for the presence of residual *E. faecalis* biofilm on the root canal walls for each group as recommended by Louwakol et al. [42].

The limitations of the current study can be the use of single-rooted teeth with single canals. Complex pulp anatomy might result in a different response of ICM to *E. faecalis*. In addition, probiotics supernatants were used for a maximum of a 7-day incubation period. Longer incubation periods and higher concentrations of probiotics supernatants may lead to a bacteria-free state. Further, this study was performed on a single type of bacterial pathogens, however, failure of RCT is usually caused by multispecies biofilm. Clinical studies with long-term follow-ups are recommended to draw a more precise conclusion on whether the multi-strain probiotics supernatants can effectively eradicate *E. faecalis* and resolve symptomatic or asymptomatic cases of AP.

## Conclusion

In single-rooted teeth with single pulp space, Ca(OH)_2_ paste was the most effective to decrease the bacterial load of *E. faecalis* after 24 h compared to MSP. However, after 7 days multi-strain probiotics supernatants were found as effective as the conventional Ca(OH)_2_ to significantly reduce the *E. faecalis* biofilm. Poloxamer hydrogel is an appropriate vehicle for MSP, however, more studies are needed to support these results and encourage its clinical application.

## Data Availability

The datasets used and/or analysed during the current study are available from the corresponding author on reasonable request.

## Abbreviations

RCT: Root canal treatment
ICM: Intracanal medication
MSP: multi-strain probiotics supernatants
BHI: Brain heart infusion
SEM: Scanning electron microscope
CFU: Colony forming unit
MRS: deMan Rosa Sharpe
CFS: cell-free supernatant
WL: Working length
EDTA: Ethylenediamine tetraacetic acid
ANOVA: Analysis of variance
